# An *Ex Vivo* Porcine Nasal Mucosa Explants Model to Study MRSA Colonization

**DOI:** 10.1371/journal.pone.0053783

**Published:** 2013-01-11

**Authors:** Pawel Tulinski, Ad C. Fluit, Jos P. M. van Putten, Alain de Bruin, Sarah Glorieux, Jaap A. Wagenaar, Birgitta Duim

**Affiliations:** 1 Department of Infectious Diseases and Immunology, Faculty of Veterinary Medicine, Utrecht University, Utrecht, The Netherlands; 2 Department of Medical Microbiology, University Medical Center Utrecht, Utrecht, The Netherlands; 3 Department of Pathobiology, Faculty of Veterinary Medicine, Utrecht University, Utrecht, The Netherlands; 4 Laboratory of Virology, Faculty of Veterinary Medicine, Ghent University, Merelbeke, Belgium; 5 Central Veterinary Institute of Wageningen UR, Lelystad, The Netherlands; University of Iowa, United States of America

## Abstract

*Staphylococcus aureus* is an opportunistic pathogen able to colonize the upper respiratory tract and skin surfaces in mammals. Methicillin-resistant *S. aureus* ST398 is prevalent in pigs in Europe and North America. However, the mechanism of successful pig colonization by MRSA ST398 is poorly understood. To study MRSA colonization in pigs, an *ex vivo* model consisting of porcine nasal mucosa explants cultured at an air-liquid interface was evaluated. In cultured mucosa explants from the surfaces of the ventral turbinates and septum of the pig nose no changes in cell morphology and viability were observed up to 72 h. MRSA colonization on the explants was evaluated followed for three MRSA ST398 isolates for 180 minutes. The explants were incubated with 3×10^8^ CFU/ml in PBS for 2 h to allow bacteria to adhere to the explants surface. Next the explants were washed and in the first 30 minutes post adhering time, a decline in the number of CFU was observed for all MRSA. Subsequently, the isolates showed either: bacterial growth, no growth, or a further reduction in bacterial numbers. The MRSA were either localized as clusters between the cilia or as single bacteria on the cilia surface. No morphological changes in the epithelium layer were observed during the incubation with MRSA. We conclude that porcine nasal mucosa explants are a valuable *ex vivo* model to unravel the interaction of MRSA with nasal tissue.

## Introduction

*Staphylococcus aureus* is an opportunistic pathogen colonizing the upper respiratory tract and skin surfaces of humans as well other mammalian species. The nose is considered to be the primary ecological niche of *S. aureus* colonization in humans [1]. Nasal carriage of *S. aureus* has been identified as a risk factor for the development of various infections in humans [1].

In 2004 a new distinct clone of methicillin-resistant *S. aureus* (MRSA) ST398 has been found in pigs in the Netherlands [2]. Since then, MRSA ST398 has been detected in pigs, veal calves and poultry around the world [3], [4]. The transmission of MRSA ST398 from livestock to humans has been reported in many countries [5], [6] and contact with livestock is recognized as a risk factor for human colonization [4], [7]. Additionally, ST398 isolates may cause infections in humans [8]. However, the mechanisms underlying successful colonization of pigs are poorly understood. Determination of the essential bacterial colonization factors is crucial to develop new treatment strategies to prevent colonization and consequently reduce MRSA ST398 interspecies transmission. Animal models are useful to study MRSA colonization. Murine [9] and rat models [10] have been developed specifically for studying *S. aureus* colonization in humans. However, the study of Gonzalez-Zorn showed that the murine nasal cavity is not a natural habitat of *S. aureus* and that this model may not be optimal to study *S. aureus* colonization [11]. Recently, *in vivo* pig colonization models have been applied [12]–[14]. Inoculation of pigs however, yielded variable results possibly due to unstable colonization [13], [14] or too low numbers of bacteria to detect with the sampling and/or isolation method used [13]. *In vivo S. aureus* colonization may be further difficult to control due to the presence of undefined local microbial and environmental factors. A suitable alternative system to gain better understanding of nasal colonization may be the use of porcine nasal mucosa explants in which bacterial and host factors can be evaluated under controlled conditions. At present there is no *ex vivo* model to study pig nasal colonization although some models based on the nasal primary tissue culture are used in virological studies [15]–[17]. In the present study we for the first time established porcine nasal mucosa explants as a novel tool to study MRSA ST398 colonization in pigs using bacterial observation of CFU changes in time or maintenance on the explants as indicators of colonization.

## Materials and Methods

### Isolation and Cultivation of the Nasal Mucosa Explants

Animals (Landrace, 6 months old sows, 70–75 kg) were MRSA negative and came from van Beek SPF pig farm B.V. (Lelystad, the Netherlands). Pigs were euthanized and exsanguinated following experimental/teaching surgery at the UMCU (Utrecht, the Netherlands). The pig head, as medical waste, was removed from the carcass, and immediately used for isolation of mucosa tissue.

Isolation of the nasal mucosa explants was performed according to the protocol of Glorieux at al. 2007 with some modifications [17]. Briefly, after removal of the head of the sow, the nose was sawed off the skull at a level just distal of the eyes. The mucosa membrane was carefully stripped from the surfaces of the ventral turbinates and septum using surgical blades (Swann-Morton No. 24), and placed in Dulbecco’s phosphate buffered saline with calcium and magnesium (DPBS) (Gibco, the Netherlands) supplemented with 1 mg/ml streptomycin (Invitrogen, the Netherlands), 1000 U/ml penicillin (Invitrogen), 1 mg/ml kanamycin (Invitrogen) and 5 µg/ml fungizone (Invitrogen).

The stripped mucosa of each tissue was divided into equal explant pieces of 1 cm^2^ using 12 mm biopsy punches (AcuDerm Inc, USA). The epithelium was placed upwards on fine-meshed gauze for culture at an air-liquid interface. The explants were cultured in serum-free medium (50% RPMI GlutaMAX™/50% DMEM GlutaMAX™ (Gibco, the Netherlands)) supplemented with 100 µg/ml gentamicin (Invitrogen), 0.1 mg/ml streptomycin and 100 U/ml penicillin (Invitrogen). The medium was added to half the height of the explant thickness to create an air liquid interface [17]. A schematic presentation of the model is shown in Figure S1. Culture was at 37°C in a 5% CO_2_ atmosphere. Ciliary beating was checked on a daily basis (each 24 h) using light microscopy as described before [17].

### Morphometric Analysis

The nasal mucosa explants were evaluated by light microscopy and by SEM after 0, 24, 48 and 72 h of cultivation. For light microscopy the explants were fixed in a phosphate-buffered 3.7% formaldehyde solution for 24 h. After fixation, the samples were embedded in paraffin. Sections (4 µm thick) were cut, deparaffinised in xylene, rehydrated in descending grades of alcohol, stained, and dehydrated in ascending grades of alcohol and xylene.

Haematoxylin–eosin staining was used to estimate the epithelial thickness. Using Soft Imaging System Leica LAS AF Lite software (Leica Microsystems, Germany) the effect of *ex vivo* culture of nasal mucosa explants on the epithelial morphometry was evaluated by measuring the epithelial thickness at five randomly selected places in five random fields.

Viability was analyzed using the ApopTag® Peroxidase *In Situ* Apoptosis Detection Kit (Chemicon, Germany) which determines DNA fragmentation. Detection is based on the terminal deoxynucleotidyl transferase mediated dUTP Nick End Labelling (TUNEL assay). Detection was performed according to the manufacturer’s instructions. TUNEL-positive cells were counted from in five randomly chosen fields of 100 cells in epithelium as well as in underlying connective tissue.

### A Microscope (Olympus Microscope BX60) was Used to Analyze All Samples at 200× Magnification

For SEM analysis explants were fixed in HEPES-buffer with 2.5% glutaraldehyde for 24 h. Afterwards, the samples were post-fixed in 1% osmium tetroxide for 2 h at room temperature. Next, the fixed samples were dehydrated in an increasing gradient of alcohol and transferred to a critical point drier. The dried samples were followed by Pt/Pd sputter coating. Cells were viewed in a field emission scanning electron microscope at 5 kV (Philips XL30S SEM FEG, Germany) at 2500× magnification.

### Bacterial Strains

Three different MRSA ST398 isolates were used to assess the ability to maintain on the nasal mucosa explants: MRSA S0462 (*spa*-type: t011, SCC*mec* IV) was isolated from a carrier pig, MRSA S0385-1 (*spa*-type: t011, SCC*mec* V) was derived from an endocarditis patient [18], whereas S0385-2 was a laboratory variant with a phage integrated in the beta-toxin gene (*hlb*), lacking the lyses of red blood cells. Additionally, human derived MRSA strain Mu50 (*spa*-type: t002, SCC*mec* II) was used. All strains were obtained from the UMCU collection (Utrecht, the Netherlands).

### Bacterial Colonization

The explants were inoculated with MRSA isolates as described previously [19]. Briefly, *S. aureus* strains were grown overnight in BHI at 37°C. A 2% aliquot was inoculated into fresh 10 ml broth and grown at 37°C under shaking (200 rpm) to mid-exponential phase (approximately 4 h). Bacteria were harvested by centrifugation at 3,750×*g* for 5 min, washed 3 times in DPBS, and suspended to an OD_600_ of 0.6 (approximately 3×10^8^ CFU/ml) in DPBS. Explants were washed with the cultivation medium without antibiotics and kept without antibiotics for at least 18 h prior inoculation. Explants were taken from the gauze and placed into a 24-well plate with the epithelial surface upwards. Next, explants were inoculated with 1 ml of bacterial suspension and allowed to adhere to the explants for 2 h at 37°C and 5% CO_2_. After incubation explants were washed three times with 1 ml of PBS and placed onto a 6-cell culture insert with 0.4 µm pores (Falcon, Becton Dickinson, the Netherlands), to prevent bacterial migration and growth in the lower chamber. To the lower chamber 3 ml of fresh cultivation medium without antibiotics was added, to the top chamber the medium was added until just a thin film of medium covered the explants (approximately 500 µl). Next, explants were cultivated for up to 3 h at 37°C and 5% CO_2_. At different post adhering time points (0, 30, 60, 90, and 180 min) of the assay the explants were washed three times in 1 ml of PBS by pipetting. Bacteria were isolated from the explants by scraping the epithelium surface using cell scrapers (Falcon, Becton Dickinson, the Netherlands), and resuspended in 1 ml of PBS with 0.1% Triton X-100. Bacterial suspensions were plated in serial dilution in PBS on Blood Agar Plates (Oxoid, UK). The plates were incubated overnight at 37°C and CFU were enumerated after 24 h. The colonization assay for each strain was repeated independently five times. Bacterial localization on the explants was determined by immunohistochemistry using mouse anti-*Staphylococcus aureus* protein A monoclonal antibody (Sigma–Aldrich, USA). At time 0 and 180 min after inoculation, explants were fixed in a phosphate-buffered 3.7% formaldehyde solution for 24 h. After fixation, the samples were embedded in paraffin. Sections (4 µm thick) were cut, deparaffinised in xylene and rehydrated in descending grades of alcohol. Next, sections were subjected to antigen retrieval by boiling in 10 mM sodium-citrate buffer (pH 6.0) and blocking of endogenous peroxidase. After rinsing in PBS/Tween20, primary antibodies (dilution 600×) were added and incubated overnight at 4°C. Sections were washed with PBS/Tween20 at RT and incubated with Powervision Goat-anti-Mouse/Rabbit/Rat IgG (Immunologic, the Netherlands) for 30 min at RT. After washing with PBS, sections were incubated with 3′,3′-diaminobenzidine (DAB) for 10 min at RT. Sections were washed with water, stained with heamatoxylin, and dehydrated in ascending grades of alcohol and xylene.

## Results

### Viability and Morphology of the Explants

The influence of cultivation on the viability and morphology of porcine nasal mucosa explants was first investigated. Viability was estimated by evaluating ciliary beating on the epithelial cells using a light microscope and by quantification of the number of apoptotic cells using the terminal deoxynucleotidyl transferase mediated dUTP Nick End Labelling (TUNEL) assay.

**Figure 1:**
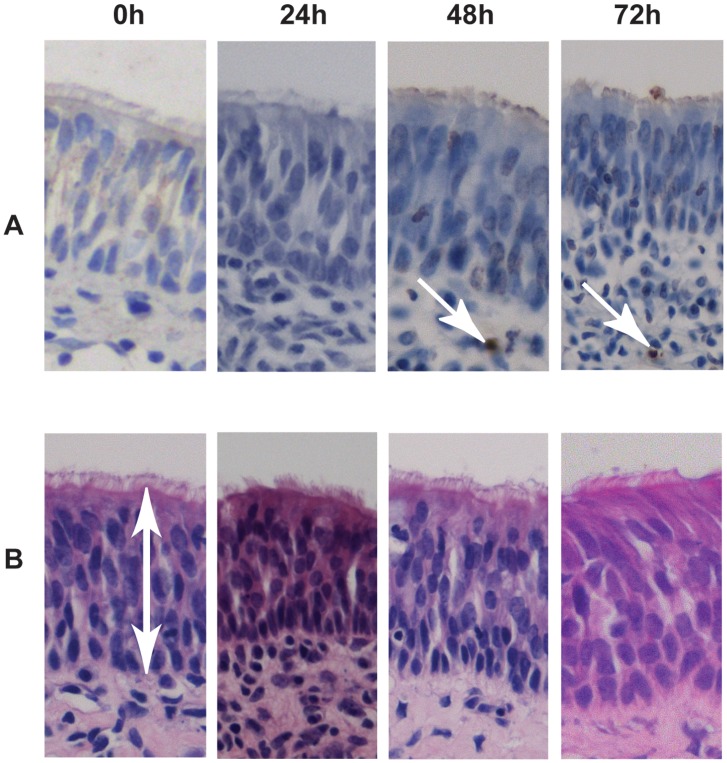
Evaluation of porcine mucosa explants after cultivation by means of light microscopy. Sections of 4 µm thickness were stained by immunohistochemistry to evaluate the apoptosis of cells. TUNEL-positive cells in the epithelium are indicated with white arrows (panel A). Panel B shows the thickness of the epithelium after staining with haematoxylin-eosin (indicated with a white arrow).

**Figure 2:**
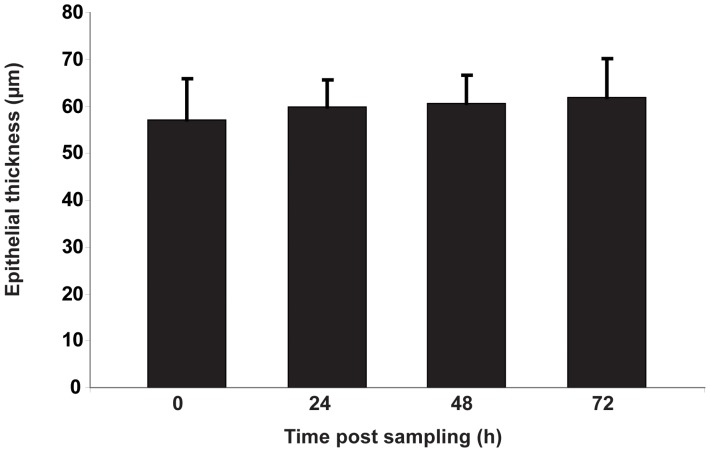
Average epithelial thickness of the porcine mucosa explants at different time points. Data are presented as mean ± standard deviation (error bars) of five independent experiment.

Cultivation of the mucosa explants for up to 72 h did not show any biological difference in the number of apoptotic cells (TUNEL-positive cells) in the epithelium (less than 1% of apoptotic cells) and basal body (less than 5% of apoptotic cells) of the explants (Figure 1A, Table 1). Morphometric analyses indicated that the explants also did not show changes in epithelial thickness after 72 h of ex *vivo* cultivation (Figures 1B and 2).

**Table 1:** Percentage of apoptotic cells as a parameter for the effect of cultivation.

Cells type	% TUNEL-positive cells at hours post sampling and cultivation			
	0	24 hours	48 hours	72 hours
Epithelium	0.0±0.0	0.0±0.0	0.4±0.2	0.6±0.5
Basal body	0.8±0.4	2.8±2.3	3.6±1.9	4.6±2.8

**Figure 3:**
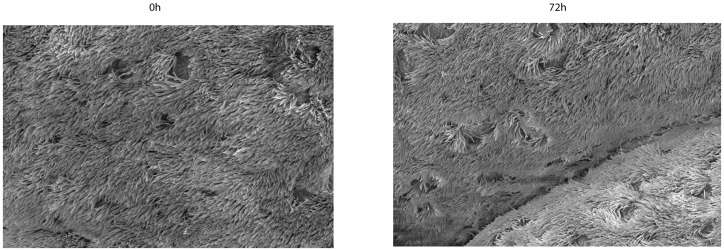
Scanning electron micrographs of porcine nasal epithelium. Epithelial cells at 0 h (A) and after 72 h (B) of *ex vivo* cultivation.

Scanning electron microscopy revealed the presence of both ciliated and non-ciliated cells at the surface of the explant. Representative scanning electron micrographs (SEM) of explants at 0 and 72 h of *ex vivo* culture illustrate ciliary cells and non-ciliary cells (Figure 3). During *ex vivo* culture, no morphological changes of the epithelium layer were observed. The cilia of the epithelial cells continued to beat up to 72 h after the start of cultivation.

### Persistence of MRSA on the Porcine Mucosa Explants

Next we investigated as to whether porcine mucosa explants could be exploited to study MRSA ST398 colonization. The ability of *S. aureus* to colonize the porcine mucosa explants was defined as persistence or outgrowth of MRSA on the explants. Three MRSA ST398 isolates were tested. One strain was isolated from a carrier pig (S0462), one human isolate originated from an endocarditis patient (S0385-1) and S0385-2 was a laboratory variant showing a different hemolysis pattern.

**Figure 4:**
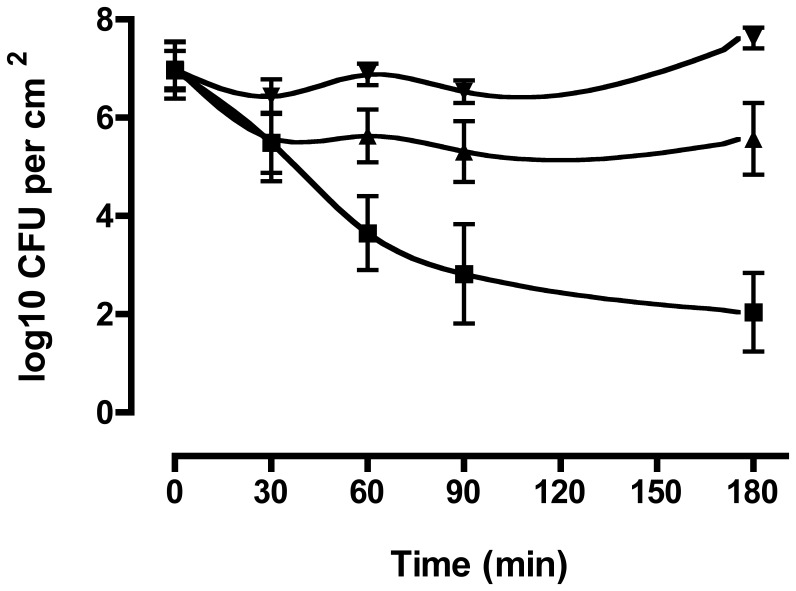
MRSA colonization of the porcine mucosa explants. Log scale presence of MRSA isolates S0462 (▾), S0385-1 (▴) and S0385-2 (▪) on the porcine nasal mucosa explants. Data are presented is the mean CFU ± standard deviation (error bars) of five different pig experiments.

Initial inoculation of the explants (1 cm^2^) was performed with 3×10^8^ colony forming units (CFU)/ml. After 2 h of incubation and washing of the explants, approximately 8×10^6^ CFU/cm^2^ (5%) adhered to the explants. The presence of the isolates on the mucosa explants was followed for an additional 180 min. During the first 30 min, S0462, S0385-1 and S0385-2 showed an initial decline in the number of CFU to approximately 3×10^6^, 2×10^5^, and 1.5×10^5^ CFU/cm^2^ respectively (Figure 4). Then, the number of recovered bacteria from isolate S0385-1 remained almost stable until the end of the experiment at approximately 4×10^5^ CFU/cm^2^. Bacterial adhesion for isolate S0462 remained stable until 90 min of the experiment. Then a significant increase to approximately 4×10^7^ CFU/cm^2^ was observed. Bacterial recovery for MRSA S0385-2 showed a gradual decline during the experiment up to approximately 2×10^2^ CFU/cm^2^ at the end of the incubation period. As a control, growth of the strains in culture medium alone without antibiotics at 37°C in 5% CO_2_ without shaking for 3 h did not show any inhibition (data not shown). These results were reproducible in five independent experiments. To verify whether the porcine nasal mucosa explants *ex vivo* model can be used to study colonization by other MRSA strains, MRSA Mu50 of human origin was used. This strain showed a similar colonization pattern as S0385-1 on the explants. The growth curve is shown in Supplementary figure S2.

**Figure 5:**
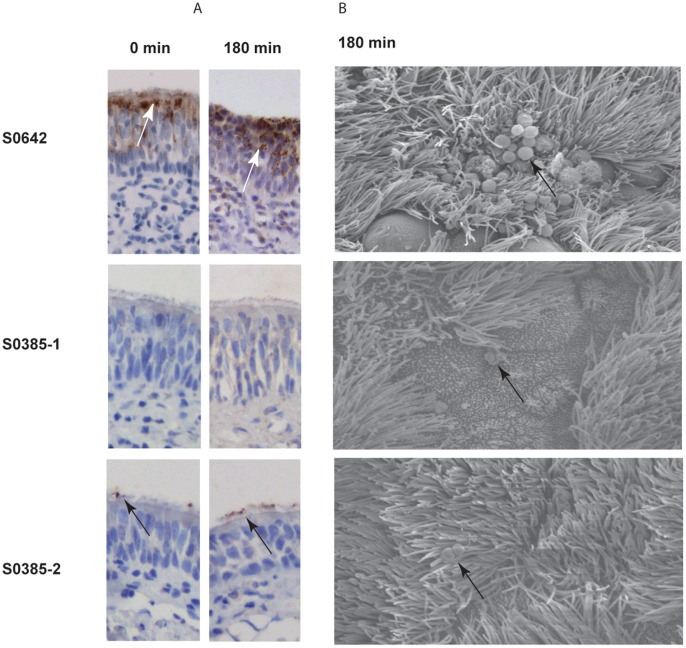
Bacterial localization on the porcine nose mucosa. Immunohistochemistry microscopy (panel A) of the porcine mucosa explants was used to determine the bacterial localization of MRSA S0462, S0385-1 and S0385-2 on explants at 0 and 180 min of colonization. Panel B shows the scanning electron micrographs of the surface of the porcine mucosa explants after 180 min colonization. Bacteria are indicated with arrows.

To visualize MRSA ST398 on the explants during the colonization assay, immunohistochemistry using anti-*Staphylococcus aureus* protein A monoclonal antibody was used (Figure 5). We were able to determine the localization of strain S0462. Isolate S0462 adhered to the surface under cilia and between epithelial cells of the top part of mucosa explants. After 180 min of incubation, the isolate S0462 formed clusters of colonies on the surface of the explants, and migration of the bacterial cells to the bottom part of the epithelium layer was observed. The immunochemistry of the explants inoculated with strains S0385-1 and S0385-2 yielded either no or a weak signal, respectively. Nevertheless, these strains showed comparable numbers of bacteria as S0462 at post adhering time 0 min. Isolate S0385-2 adhered and remained on to the surface of the cilia itself. After 180 min of incubation with bacteria cells, isolate S0462 showed clustering of bacteria on the surface of the explants and further migration of the bacteria in the epithelium layer. Isolate S0385-2 remained on the surface of the cilia. To visualize the interaction of the bacteria with the epithelium in more detail, SEM was performed on the explants at 180 min of the incubation. Bacteria of the strain S0462 were present as clusters on the epithelial surface between the cilia (Figure 5, panel B). Strain S0385-2 remained on the cilial surface and did not appear in clusters. SEM on bacteria of the S0385-1 showed bacteria located on the epithelial surface. Interaction of MRSA ST398 isolates with the tissue did not result in visible changes in morphology of the inoculated epithelium.

## Discussion

We evaluated porcine nasal mucosa explants as a model system to study *S. aureus* colonization in pigs. The model was adapted from a study on interaction of viruses with the respiratory tract [17]. *In vitro* adhesion of *S. aureus* to monolayer cell cultures have been used, especially to study bacterial interaction with human cells [19]. The limitation of this system is the lack of diversity in cell types and often the lack of the presence of mucus [20]. The nasal environment contains different types of epithelial cells [21]. The *ex vivo* explants model system applied here was designed to overcome these limitations and to better display the many characteristics and cell types of the porcine nasal mucosa cells *in vivo*.

The porcine nasal mucosa explants were cultivated at an air-liquid interface which creates a physiological environment corresponding to natural conditions. As described earlier [17], serum-free medium was used to cultivate the explants. It has been reported that use of fetal calf serum results in enlarged epithelial cells, loss of cell–cell contacts and a loose epithelium. With the conditions employed in our hands, we successfully maintained porcine nasal respiratory explants for at least 72 h without any signs of gross changes in cell viability as measured by the presence of apoptotic cells (more than 5%). Similarly, morphometric analyses of the mucosa explants showed no major changes during *ex vivo* cultivation. Furthermore, ciliary beating was observed during the entire cultivation and SEM showed that cell-cell contact and three-dimensional structures of the tissue were preserved. Together, these results indicate that the porcine nasal mucosa explants preserved their integrity and viability in *ex vivo* conditions for up to 72 h under the conditions employed.

To evaluate porcine nasal mucosa explants as a new tool to study MRSA colonization in pigs, we used three MRSA ST398 strains as inoculum. All showed reproducible adherence to the epithelial layer of the mucosa explants. However, we observed differences in persistence of the isolates. One isolate was from a carrier pig (S0462), the two other strains were from a patient with endocarditis (S0385-1 and S0385-2). All three isolates showed an initial decline in the number of CFU during the first 30 min after inoculation which might indicate bacteria adaptation to the explants. After 30 min of post adhering period, bacterial recovery from the experiments showed a significant increase of the number of CFU for isolate S0462, unaltered bacterial number for isolate S0385-1, and a loss bacteria for isolate S0385-2, suggesting differences in interaction of the different isolates with the tissue. Additionally, the colonization assay using a human derived MRSA strain Mu50 showed a similar pattern to MRSA S0385-1. We conclude that this model can be use to study MRSA colonization belonging to different clonal complexes and human origin.

Attempts to visualize the bacteria on the tissue was performed using anti-*Staphylococcus aureus* protein A monoclonal antibodies were only partially successful. Isolates S0385-1 and S0385-2 were probably poorly visible due to the presence of low numbers of bacteria and/or insufficient expression of protein A. It has previously been documented that some *S. aureus* strains show no or very low expression of protein A *in vivo* [22]. Additionally, Western blotting of stationary grown bacteria in BHI medium confirmed poor expression of protein A in these strains (data not shown). SEM of tissue carrying isolate strain S0462 revealed clusters of bacteria located between the cilia. For the two isolates S0385-1 and S0385-2 only single bacteria were observed. The absence of bacterial clusters may be caused by the lower number of bacteria recovered for these strains. However, we cannot exclude the alternative possibility that the lack of bacterial cluster formation contributed to the poor bacterial recovery of these isolates. Our observations do suggest that different MRSA isolates display variable qualities in colonizing mucosa explants, which perhaps mimics natural host colonization. Due to the fact that adhesion of the bacteria to the tissue was performed in DPBS, which is not reflecting the *in vivo* situation, small changes comparing with *in vivo* situation may occur.

A major advantage of the *ex vivo* porcine nasal mucosa explants model is that different bacterial strains can be tested under controlled conditions. From one animal around 20 explants (1 cm^2^) can be obtained and multiple strains can be tested simultaneously eliminating genetic variation of the host. In addition, the model can be readily adapted for other bacterial species or the introduction of multiple species. The successful establishment of porcine nasal mucosa explants to study of the interaction of *S. aureus* isolates with nasal tissue enables studies to better understand the mechanisms of colonization of MRSA in pigs and may aid future assessment of the effects of potential inhibitory compounds on this process.

## Supporting Information

Figure S1 **Schematic cross-section of a culture system using nasal mucosa explant with an air-liquid interface.**
(TIF)

Figure S2 **Log scale presence of pig origin MRSA S0462 and the human derived strain Mu50 on the porcine nasal mucosa explants.** Data are presented as mean CFU ± standard deviation (error bar) of five different pig experiments. MRSA S0462 belongs to ST398 *spa*-type: t011 SCC*mec* V. MRSA Mu50 belongs to CC5 *spa*-type t002 SCC*mec* II. Strain Mu50 shows successful colonization on the porcine nasal mucosa explants, although variation between experiments was bigger with Mu50 compared to S0462.
(TIF)
