# Supplementary figures and images for: An Ex Vivo Porcine Nasal Mucosa Explants Model to Study MRSA Colonization
Source: PLoS One. 2013 Jan 11;8(1):e53783. doi: 10.1371/journal.pone.0053783 (PMC3543263; doi:10.1371/journal.pone.0053783)

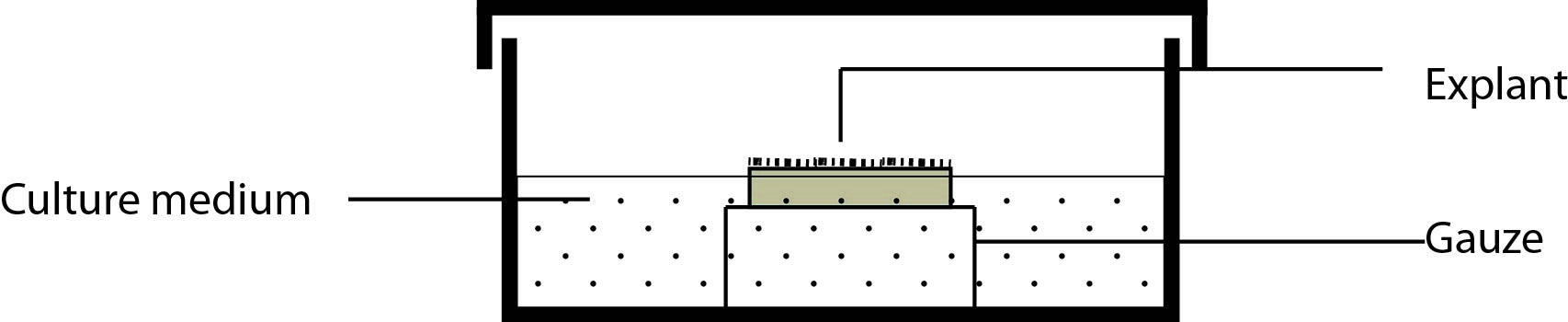

Supplement: Figure S1 — Schematic cross-section of a culture system using nasal mucosa explant with an air-liquid interface. (TIF) [file pone.0053783.s001.tif]

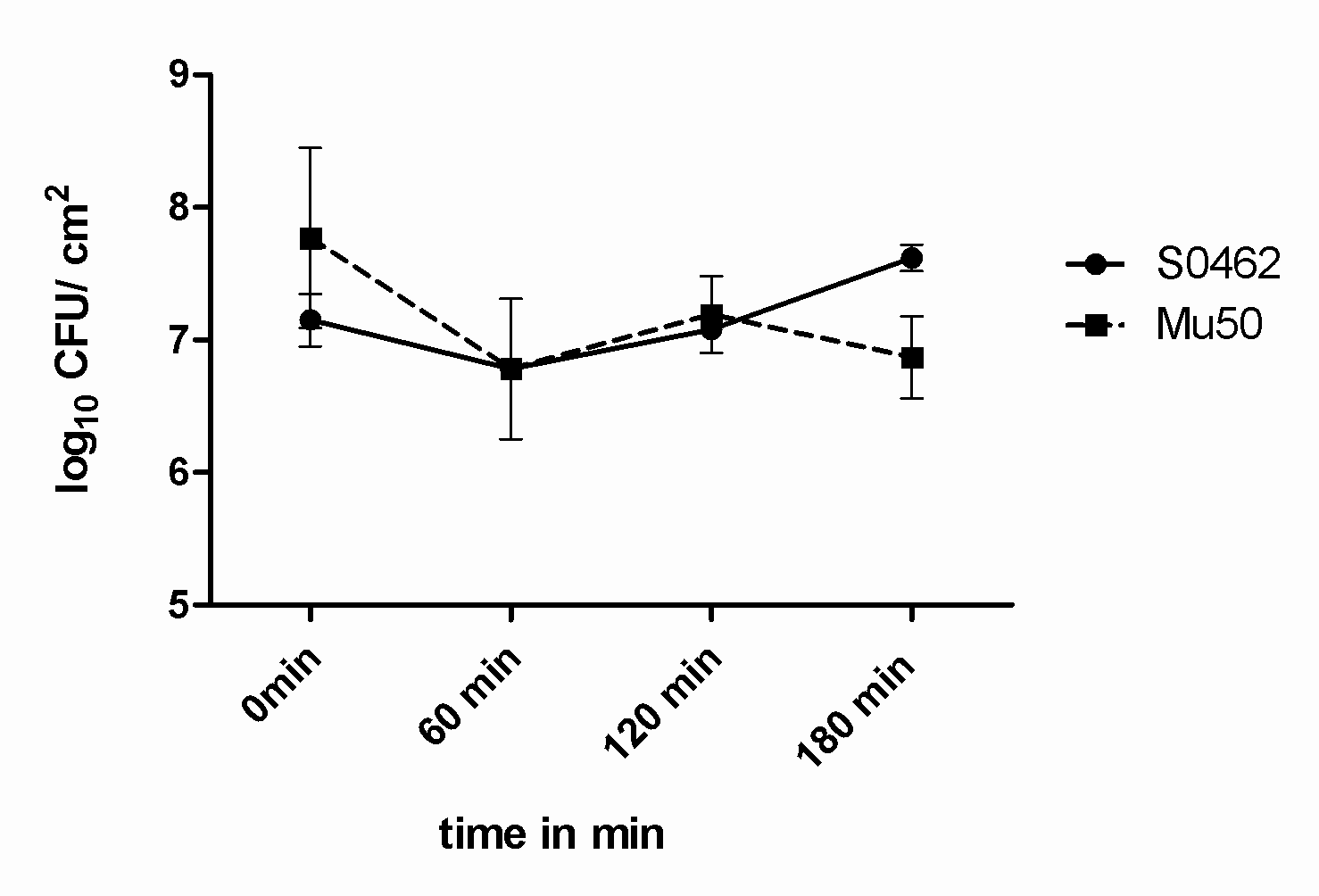

Supplement: Figure S2 — Log scale presence of pig origin MRSA S0462 and the human derived strain Mu50 on the porcine nasal mucosa explants. Data are presented as mean CFU ± standard deviation (error bar) of five different pig experiments. MRSA S0462 belongs to ST398 spa-type: t011 SCCmec V. MRSA Mu50 belongs to CC5 spa-type t002 SCCmec II. Strain Mu50 shows successful colonization on the porcine nasal mucosa explants, although variation between experiments was bigger with Mu50 compared to S0462. (TIF) [file pone.0053783.s002.tif]
